# Single extracellular vesicle analysis in human amniotic fluid shows evidence of phenotype alterations in preeclampsia

**DOI:** 10.1002/jev2.12217

**Published:** 2022-05-18

**Authors:** Natalia Gebara, Julia Scheel, Renata Skovronova, Cristina Grange, Luca Marozio, Shailendra Gupta, Veronica Giorgione, Federico Caicci, Chiara Benedetto, Asma Khalil, Benedetta Bussolati

**Affiliations:** ^1^ Department of Molecular Biotechnology and Health Sciences University of Turin Turin Italy; ^2^ Department of Systems Biology and Bioinformatics University of Rostock Rostock Germany; ^3^ Department of Medical Sciences University of Turin Turin Italy; ^4^ Department of Surgical Sciences, Obstetrics and Gynecology, University of Turin Turin Italy; ^5^ Vascular Biology Research Centre Molecular and Clinical Sciences Research Institute St George's University of London London UK; ^6^ DiBio Imaging Facility University of Padua Padua Italy; ^7^ Foetal Medicine Unit St George's University Hospitals NHS Foundation Trust St George's University of London London UK

**Keywords:** amniotic fluid, angiogenesis, exosomes, extracellular vesicles, placenta, pregnancy disorders, soluble endoglin

## Abstract

Amniotic fluid surrounding the developing fetus is a complex biological fluid rich in metabolically active bio‐factors. The presence of extracellular vesicles (EVs) in amniotic fluid has been mainly related to foetal urine. We here characterized EVs from term amniotic fluid in terms of surface marker expression using different orthogonal techniques. EVs appeared to be a heterogeneous population expressing markers of renal, placental, epithelial and stem cells. Moreover, we compared amniotic fluid EVs from normal pregnancies with those of preeclampsia, a hypertensive disorder affecting up to 8% of pregnancies worldwide. An increase of CD105 (endoglin) expressing EVs was observed in preeclamptic amniotic fluid by bead‐based cytofluorimetric analysis, and further confirmed using a chip‐based analysis. HLA‐G, a typical placental marker, was not co‐expressed by the majority of CD105^+^ EVs, in analogy with amniotic fluid stromal cell derived‐EVs. At a functional level, preeclampsia‐derived EVs, but not normal pregnancy EVs, showed an antiangiogenic effect, possibly due to the decoy effect of endoglin. Our results provide a characterization of term amniotic fluid‐EVs, supporting their origin from foetal and placental cells. In preeclampsia, the observed antiangiogenic characteristics of amniotic fluid‐EVs may reflect the hypoxic and antiangiogenic microenvironment and could possibly impact on the developing fetus or on the surrounding foetal membranes.

## 1 INTRODUCTION

Amniotic fluid is a complex biological fluid surrounding the developing fetus and in close proximity to the placenta (Underwood et al., 2005). Its composition, after foetal skin keratinization, mainly consists of foetal cardiovascular, renal, pulmonary, and endothelial secretions (Roubelakis et al., 2012). Amniotic fluid has a relevant role in foetal physiology; it not only acts as a protective cushion to mechanical injury but also contains a plethora of active factors, including nutrients, growth and antimicrobial factors, cells and extracellular vesicles (EVs) (Underwood et al., 2005).

EVs are a highly heterogeneous population of small particles enclosed in a lipid bilayer, released by almost all cell types, and involved in cell‐to‐cell signalling. In pregnancy, EVs are essential for diverse physiological and pathological processes (Jankovičová et al., 2020) and may play a role in angiogenesis and successful foetal development (Gebara et al., 2021). The role of EVs derived from placental trophoblasts has been studied extensively, as they are continuously released into the maternal bloodstream and play a crucial role in regulating the maternal immune response and pregnancy adaptation (Nair & Salomon, 2018). At variance, the profile and function of EVs present in amniotic fluid have been poorly investigated, as their characteristics have been mainly attributed to foetal urine production (Keller et al., 2007). Indeed, previous studies showed that a large proportion of EVs within the amniotic fluid express typical renal markers, such as aquaporin‐2 and CD24, demonstrating their foetal urine origin (Keller et al., 2007). However, multiple cells may release EVs into the amniotic fluid, including the amnion, placental tissues and foetal cells (Dixon et al., 2018; McMaster et al., 1998). Interestingly, isolated amniotic epithelial or stem cells in culture were shown to release EVs with trophic, immunomodulating and anti‐inflammatory functions (Balbi et al., 2017; Sedrakyan et al., 2017). It is therefore conceivable that EVs released from those cells are also present in the amniotic fluid. Moreover, a recent trial reported the safe administration of a pharmaceutical product, including term amniotic fluid‐EVs, against the COVID‐19 related cytokine storm (Mitrani et al., 2021). The analysis and characterization of amniotic fluid EVs is therefore of interest.

Preeclampsia is a complex pregnancy disorder characterized by new‐onset hypertension with associated maternal organ dysfunction, including impaired liver function, renal damage (significant proteinuria, oligoanuria, increased creatinine), low platelets, haemolysis, decreased sPO_2_ or pulmonary edema, or visual and cerebral disturbances (Brown et al., 2018). Preeclampsia affects up to 8% of pregnancies world‐wide (Duley, 2009) and causes significant maternal and perinatal morbidity and mortality, as well as long‐term complications (Amaral et al., 2015). Abnormal placentation and insufficient trophoblast invasion are considered the main causes of preeclampsia (Kaufmann et al., 2003), leading to the release of antiangiogenic factors that subsequently promote maternal endothelial dysfunction (Staff et al., 2007). Several studies highlighted the presence of altered EVs in the maternal circulation of preeclamptic women and their possible contribution to endothelial dysfunction (Escudero et al., 2016). Interestingly, EVs carrying antiangiogenic factors, such as soluble vascular endothelial growth factor receptor 1 (sFlt‐1) and soluble endoglin, were found at high levels in the maternal circulation and were shown to be released by placental explants (Chang et al., 2018; Nair et al., 2021; Tannetta et al., 2013). Therefore, it is of interest to evaluate whether amniotic fluid EVs in preeclamptic pregnancies might have phenotypic or functional differences with respect to those of normal pregnancies.

In the present study, we aimed to characterize at phenotypic and functional level amniotic fluid‐derived EVs from term normal and preeclamptic pregnancies.

## 2 MATERIALS AND METHODS

### 2.1 Collection of amniotic fluid

Amniotic fluid samples (40 from normal pregnancies and seven from preeclamptic pregnancies) were collected during caesarean delivery in the Department of Surgical Sciences at the University of Turin, after approval by the Ethics Review Board of the Health and Science City of Turin, (Città della Salute e della Scienza di Torino, protocol N° 0079734, CS2/320) and at the Maternity Department of St George's University Hospital in London, after approval by the Local Ethics Committee (19/LO/0794) (Table 1). All patients provided preoperative written informed consent. The fluid was collected by sterile acupuncture after myometrial incision, before the incision of the amniotic membranes. Three of the preeclamptic patients (*n* = 7) were affected by comorbidities, including foetal growth restriction, type two diabetes, and systemic lupus erythematosus. Further clinical information about preeclamptic patients can be found in Table 2.

**TABLE 1:** Clinical information showing the mean ± SD of gestational age, maternal age at delivery and the volume of the amniotic fluid obtained from normal and preeclamptic pregnancies

	Normotensive pregnancies (*n* = 40)	Preeclampsia (*n* = 7)
Gestational age (weeks)	37.9 ± 1.8	36.1 ± 2.6
Maternal age (years)	35.7 ± 5.2	32.8 ± 6.7
Amniotic fluid volume (ml)	19.9 ± 7.8	18.4 ± 11.3

**TABLE 2:** Clinical information on individual preeclamptic patients showing the gestational age, maternal age at delivery, week of preeclampsia diagnosis, sFlt‐1/PIGF ratio, other pathologies, treatment, and additional relevant information. PIGF:Placental growth factor

	Age of gestation (weeks + days)	Maternal age (years)	Preeclampsiadiagnosis (weeks)	sFlt‐1/PIGF ratio	Other pathologies	Treatments	Other
1	36 + 1	42	36	280	–	Betamethasone Nifedipine	Assisted reproduction techniques
2	32 + 1	32	31	531	–	BetamethasoneNifedipine MgSO_4_	Foetal growth restriction
3	39 + 2	22	36	42	–	–	–
4	36 + 6	39	35	–	Chronic hypertension, systemic lupus erythematosus	Methyldopa, Nifedipine, Hydroxychloroquine, Aspirin, Low Molecular Weight Heparin	–
5	38 + 2	28	36	–	Type 2 diabetes	Metformin, Insulin	–
6	36 + 1	35	36	–	–	Labetalol, Nifedipine	Foetal growth restriction
7	36 + 2	33	33	–	–	Labetalol, Nifedipine, Metformin, Insulin	–

### 2.2 EV isolation from amniotic fluid

Amniotic fluid samples were diluted in PBS up to a volume of 20 ml and subjected to differential centrifugations. For the isolation of cells, samples underwent a 5 min centrifugation at 300 × *g*, at room temperature. A second centrifugation of 10 min was performed at 500 × *g*, at room temperature, for removal of debris. The supernatant was then passed through a cell sieve and underwent a 30 min ultracentrifugation (70 Ti rotor, Beckman Coulter, CA, USA) at 10,000 × *g*, at 4°C. The resulting pellet was discarded, and the supernatant was further centrifuged for 2 h at 100,000 × *g* at 4°C. The final step included resuspension of EV pellets in 1 ml 1% DMSO/PBS and additional filtration through a 0.22 μm filter. The recovered EVs were stored at −80°C.

### 2.3 Isolation and culture of term amniotic fluid mesenchymal stromal cells (AFSCs)

Term AFSCs were previously obtained and characterized, as described (Iampietro et al., 2020). Briefly, amniotic fluid was transferred into a sterile 50 ml falcon and centrifuged at 500 × *g* for 10 min at room temperature. The cell pellet was resuspended in α‐MEM Medium (Gibco/BRR, ThermoFisher, MA, USA) supplemented with 20% Chang Medium B (Irvine Scientific, Santa Ana, California, USA) and 2% Chang Medium C (Irvine Scientific), 20% Foetal Calf Serum (Invitrogen, Carlsbad, CA, USA), 50 IU/ml penicillin, 50 g/ml streptomycin, 5 mM glutamine (all from Sigma‐Aldrich, St. Louis, MO, USA). The cells were placed into a T25 flask, incubated at 37°C on 5% CO_2_ until the cells formed clusters (1–2 weeks). Afterwards, the medium was replaced twice a week until the cells reached confluency. Under these culture conditions, AFSCs expressed typical mesenchymal surface markers and differentiation properties, as previously described (Iampietro et al., 2020).

### 2.4 EV isolation from AFSCs

The term AFSCs were grown to 70–80% confluency, washed twice with PBS and placed in serum free RPMI medium overnight. After 16 h of incubation, the RPMI medium was recovered and centrifuged at 300 × *g* for 5 min, 10,000 × *g* for 30 min, followed by ultracentrifugation at 100,000 × *g* for 2 h at 4°C. The EV pellet was resuspended in 1% DMSO/PBS and stored at −80°C.

### 2.5 Smart SEC purification

Size‐exclusion chromatography was applied to samples destined for miRNA arrays and ExoView analysis. Following differential ultracentrifugation, EV pellets were resuspended in 100 μl of PBS and applied to Smart SEC columns (SSEC100A‐1, BioNova, Madrid, Spain). The samples were then processed as recommended by the manufacturer's protocol.

### 2.6 Transmission electron microscopy

One drop of PBS (about 25 μl) containing EV at 5.8 × 10^8^ was placed on a 400‐mesh holey film grid; after staining with 2% uranyl acetate (for 2 min) the sample was observed with a Tecnai G2 transmission electron microscope (FEI Company, Hillsboro, OR, USA) operating at 100 kV. Images were captured with a Veleta digital camera (Olympus Soft Imaging System, Tokyo, Japan).

### 2.7 Nanosight analysis

The EV size and concentration was determined by nanoparticle tracing analysis (NTA) using the NanoSight LM10 system (Malvern Panalytical, Malvern, UK). Each sample was diluted (1:100) in PBS (filtered with a 0.22 μm filter). For each sample, a syringe pump flow of 30 was applied. Three videos of 60 s each were recorded and analysed, calculating an average number of EV size and concentration (particles/ml). All samples were characterized with NTA 3.2 analytical software.

### 2.8 Super‐resolution microscopy

Super‐resolution microscopy pictures of EVs were obtained using a temperature‐controlled Nanoimager S Mark II microscope from ONI (Oxford Nanoimaging, Oxford, UK) equipped with a 100×, 1.4NA oil immersion objective, an XYZ closed‐loop piezo 736 stage, and dual or triple emission channels split at 640 and 555 nm. For sample preparation we followed the manufacturer's protocol. Then, the anti‐CD9 conjugated with Atto 488 dye (ONI), anti‐CD63 antibodies (SC‐5275, SC‐31234, Santa Cruz, CA, USA) conjugated with Alexa Fluor 647 dye, HLA‐G antibody (SC‐21799, Santa Cruz) conjugated with Alexa Fluor 555 dye, were prepared using the Apex Antibody Labelling Kit (Invitrogen) according to the manufacturer's protocol as described (Skovronova et al., 2021). Images were taken in dSTORM mode acquired sequentially in total reflection fluorescence (TIRF) mode. Single‐molecule data was filtered using NimOS software (v.1.18.3, ONI). Data has been processed with the Collaborative Discovery (CODI) online analysis platform www.alto.codi.bio from ONI and the drift correction pipeline version 0.2.3 was used.

### 2.9 Western blot

For protein analysis, EVs were lysed at 4°C for 30 min in RIPA buffer (20 nM Tris‐HCl, 150 nM NaCl, 1% deoxycholate, 0.1% SDS, 1% Triton X‐100, pH 7.8) supplemented with a cocktail of proteases, phosphatase inhibitors and PMSF (Sigma‐Aldrich). As determined by the Bradford method, aliquots of the cell lysates containing 10–15 μg of proteins were run on 4–20% acrylamide gel SDS‐PAGE under reducing conditions. The transfer of proteins onto a PVDF membrane was performed using the iBlot™ Dry Blotting System (Life Technology). Primary antibodies used were: CD81 at 1:200 (SC‐31234), CD63 at 1:200 (SC‐5275), HLA‐G at 1:200 (SC‐21799), (all from Santa Cruz), CD105 at 1:1000 (362‐820, Ancell) and at 1:2000 calreticulin (2891S, Cell Signaling Technology, Danvers, MA, USA). Chemiluminescent signal was detected using the ECL substrate (Bio‐Rad), and bands were detected by the ChemiDoc system (Bio‐rad, Hercules, CA, USA). 

### 2.10 MACSPlex

EV samples were tested with a bead‐based multiplex analysis (MACSPlex exosome kit, human, Miltenyi Biotec, Bergisch Galdbach, Germany). EV samples were diluted with MACSPlex buffer to a final volume of 120 μl with 5.8 × 10^8^ EVs per sample and processed following the manufacturer's protocol. Median fluorescence intensity was calculated for all 39 bead populations. The analysis of the samples was performed by subtraction of the control and the fluorescence controls. For analysis of AFSC‐EVs, the fluorescent antibody mix against tetraspanins (CD63, CD81 and CD9) was substituted with the APC labelled HLA‐G antibody (130111852, Miltenyi Biotec, same volume of 15 μl). This method ensured that EVs attached to the Ab conjugated beads were only detected if positive for HLA‐G.

### 2.11 ExoView array

For the analysis with the ExoView platform™ (NanoView Biosciences, Boston, MA, USA), EVs were further purified with Smart SEC columns (BioNova), and the collected fractions were applied directly to the ExoView chips following the manufacturer's protocol. Alexa Fluor 488 conjugated anti‐CD117 (ab2164459, Abcam, Cambridge, UK), anti‐Tie‐2 (ab24859, Abcam), anti‐CD105 (362‐820, Ancell) and HLA‐G conjugated with Alexa Fluor 555 (SC‐21799, Santa Cruz) were used for detection of EV subpopulations. For all antibodies, the Apex Antibody Labelling Kit was used (Invitrogen). All antibody conjugations were performed according to manufacturer's protocol. Chips were analysed using the ExoView™ R100 reader using ExoView Scanner software (v 3.0). The data were analysed using ExoView Analyzer (v 3.0).

### 2.12 Angiogenesis

Human umbilical vascular endothelial cells (HUVEC) were bought from ATCC (ATCC‐PCS‐100‐010, Manassas, VA, USA) and cultured until the 6th passage in EBM medium (Lonza, Basel, Switzerland). In vitro formation of capillary‐like structures was performed on growth factor–reduced Matrigel (356231, Corning, NY, USA). HUVEC cells were treated with EVs (1000 EVs/cell), anti‐CD105 TRC‐105 Ab (8 μg/ml, TRACON Pharmaceuticals, CA, USA) and recombinant soluble CD105 (s‐Endoglin, 100 ng/ml; C‐60059, PromoKine, Heidelberg, Germany) in EBM medium and seeded at a density of 15 × 10^3^ cells/well on a 24‐well plate. For combination of EVs with the anti‐CD105 Ab TRC‐105, both components were mixed and incubated for 25 min at room temperature, prior to adding them to the cell suspension. Cells were periodically observed with a Nikon TE2000E inverted microscope (Nikon, Tokyo, Japan), and experimental results were recorded after 16 h; five images were taken per well. Image analysis was performed with the ImageJ software v.1.53c, using the Angiogenesis Analyzer. The data from three independent experiments were expressed as the mean ± SD of tube length in arbitrary units per field.

### 2.13 Statistical tests

All statistical analyses were performed using GraphPad Prism software (v. 8.00; GraphPad, CA, USA). For comparison of each group, an unpaired Student's *t*‐test was used. FWelch's correction was performed when the variance across groups was assumed to be unequal. Differences were considered significant when the *p*‐value was <0.05. For multiple comparison analyses, ANOVA with Bonferroni multicomparison post hoc test. A two‐tailed *p*‐value <0.05 was considered statistically significant.

## 3 RESULTS

### 3.1 Characterization of amniotic fluid‐derived EVs

**FIGURE 1:**
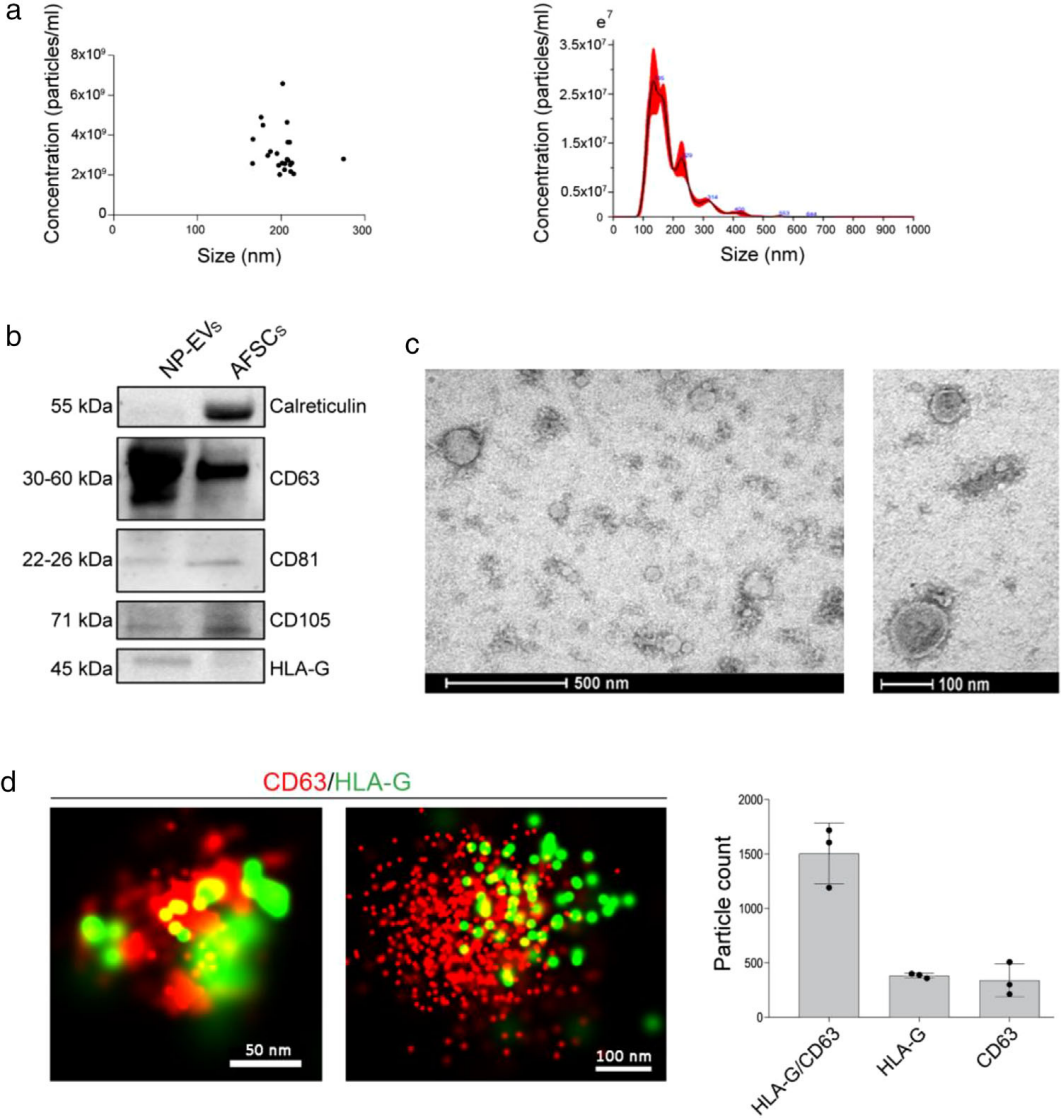
Characterization of NP‐EVs. (a) Nanoparticle tracking analysis of NP‐EVs (*n* = 30) showing homogenous size and concentration. Dilution of 1:100 in PBS in 1ml was used to test the samples (left panel). A representative graph showing size distribution of a NP‐EV sample (right panel). (b) Representative Western blot image showing the presence of CD63, CD81, CD105 and HLA‐G in NP‐EVs (10–15 μg proteins) and in AFSC lysate (10–15 μg proteins) and the presence of calreticulin in cell lysate only. Three different NP‐EV samples were tested with similar results. (c) Representative transmission electron microscopy images at low‐ and high‐power fields of NP‐EVs showing heterogenous EV population. (d) Representative super‐resolution microscopy images of single amniotic fluid‐derived EVs expressing CD63 (red) and HLA‐G (green). The number of single and double positive EVs for CD63 and HLA‐G was analysed in three NP‐EV preparations using the CODI software; the graph shows the mean ± SD of a cumulative analysis of 10 fields for each preparation, total EV number: 6676

EVs were isolated from term amniotic fluid of 40 normal pregnancies (Table 1) by differential ultracentrifugation followed by filtration and, for selected experiments (miRNA and ExoView analysis), by size‐exclusion chromatography. The normal pregnancy EVs (NP‐EVs) were first characterized based on their size and concentration using nanoparticle tracking analysis (Figure 1a). Homogeneous concentration and particle size were obtained among the different samples, with a mean size of 221.8 ± 6.2 nm. Western Blot analysis confirmed the expression of EV specific markers (CD63, CD81) and revealed the presence of the placental maker HLA‐G, previously shown to characterize placental‐derived EVs (Orozco et al., 2009) (Figure 1b). The absence of the cytoplasmic marker calreticulin showed a lack of cellular contamination (Figure 1b). TEM showed heterogeneous EV populations (particles ranging from approximately 50 to approximately 200 nm in diameter) (Figure 1c). To quantify the expression of CD63 and HLA‐G and to assess their possible colocalization, we performed super‐resolution imaging of NP‐EVs using dSTORM. More than 6000 single‐EV images were acquired and analysed. Clustering analysis showed that the majority (around 87%) of CD63^+^ EVs also expressed HLA‐G (Figure 1d).

**FIGURE 2:**
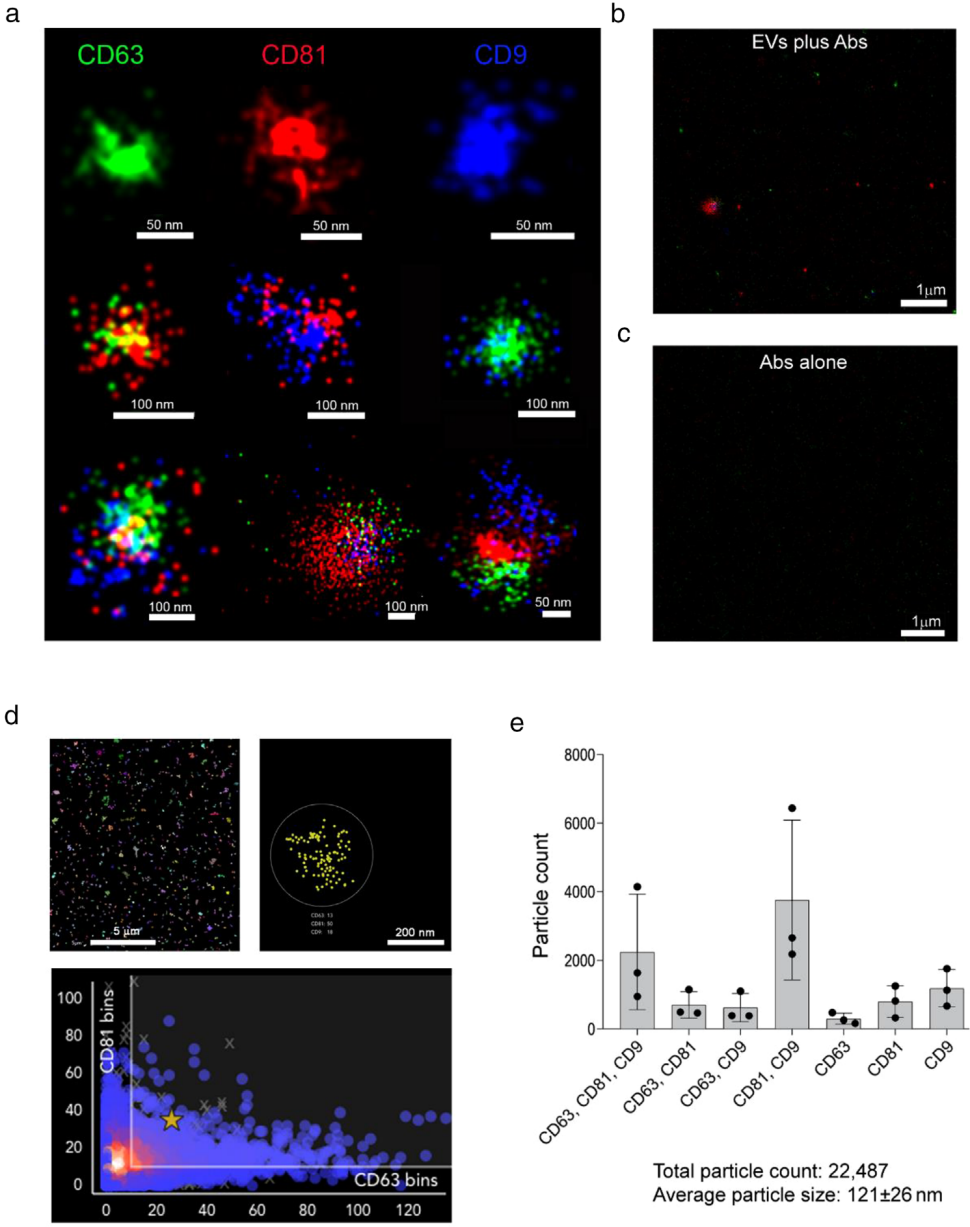
Super‐resolution microscopy analysis of tetraspanins expression in NP‐EVs. (a) Representative super‐resolution microscopy images of NP‐EVs showing single, double, and triple expression of CD63 (green), CD81 (red), CD9 (blue). The corresponding scale bare is below each EV image. (b) Representative super‐resolution images, at low magnification, showing staining with anti‐tetraspanin antibodies in the presence of EVs (EVs plus Abs) or (c) in the absence of EVs (Abs alone). (d) Representative clustering strategy of NP‐EV analysis showing a large field of view (left panel), a selected cluster (right panel) and a graph (bottom panel) of CD81/CD63 cluster distribution. (e) Clustering analysis of super‐resolution microscopy images showing the single, double, and triple positive EV fractions expressing the tetraspanin markers. The analyses were performed in three NP‐EV preparations using a CODI software; the graph shows the mean ± SD of a cumulative analysis of 10 fields for each preparation

In addition, super‐resolution microscopy was used to analyze the size and expression of tetraspanin markers (CD63, CD81 and CD9) at a single‐molecule level in NP‐EVs (Figure 2). The results showed that NP‐EVs were a heterogeneous population with highly variable tetraspanin expression (Figure 2). The analysis of more than 20,000 EVs using a dedicated software (https://alto.codi.bio/) showed that the majority of EVs were either triple positive for CD63, CD81 and CD9 or double positive for CD81 and CD9, with lower fractions expressing double or single tetraspanins (Figure 2e). The overall average particle size evaluated by the super‐resolution microscope was 121 ± 26 nm. This measurement, based on specific EV detection using tetraspanins, overlaps the results obtained by transmission electron microscopy. The larger EV size detected by nanoparticle tracking analysis can be possibly explained by the evaluation of the EV hydrodynamic size, under solution using this technique, in comparison with the measurement of the dry radius with microscopy techniques (Montis et al., 2017). In addition, possible artifacts including EV aggregates or non‐EV particles can be detected by nanoparticle tracking analysis, suggesting that it should be applied for size evaluation together with other side techniques, as previously described (Skovronova et al., 2021).

### 3.2 Comparison of amniotic fluid EVs from normal and preeclamptic pregnancies

**FIGURE 3:**
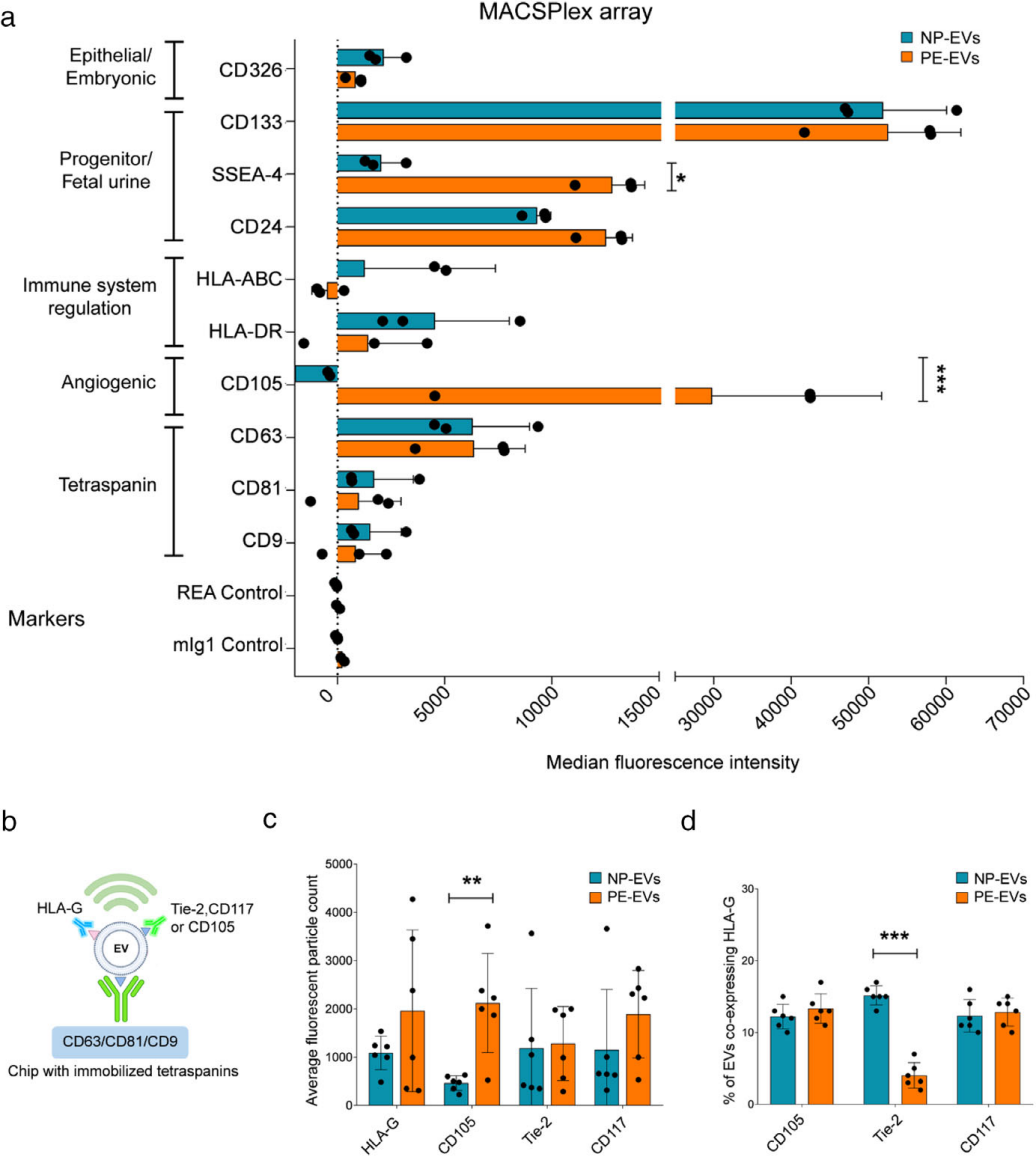
Increased CD105 expression in PE‐EVs. (a) MACSPlex analysis showing the median fluorescence intensity of surface markers characteristic of EVs (tetraspanins) or different cell of origin, expressed by NP‐ and PE‐EVs (*n* = 3). Expression of CD105 and SSEA‐4 was significantly increased in PE‐EVs. 5.8 × 10^8^ EVs were analysed, diluted to a final volume of 120 μl of MACSPlex buffer. (b) Diagram explaining the experimental method behind ExoView technology in relation to the graphs in panel c. (c and d) ExoView analysis of amniotic fluid‐derived NP‐EVs (*n* = 6) and PE‐EVs (*n* = 6). (c) Comparison of the expression of HLA‐G, Tie‐2, CD105 and CD117 (c‐kit) shown as average fluorescent particle count in NP‐EVs vs PE‐EVs from combined tetraspanins capture of CD63, CD81 and CD9. (d) Normalized expression of HLA‐G positive EVs co‐expressing other angiogenic (CD105 and Tie‐2) and stem cell (CD117) markers. 5.8 × 10^8^ EVs in final volume of 35 μl of buffer were used for all samples. Unpaired student's *t*‐test:* = *P* < 0.05, * * = *P* < 0.01, *** = *P* < 0.001

EVs were also isolated from term‐amniotic fluid of 7 preeclamptic pregnancies (Table 1). The different volumes and EV concentrations in each sample are reported in Supplementary Table 1. No difference in mean EV concentration between NP‐EVs and PE‐EVs (2.85 × 10^9^ and 2.17 × 10^9^ particles/ml, respectively) was observed, although the relevance of this parameter is limited by the dynamic nature of this biofluid (Underwood et al., 2005). As amniotic fluid contains EVs originating from several sources, a comprehensive analysis of EV surface receptors characterizing several cell types was performed using MACSPlex, a bead‐based cytofluorimetric kit able to perform a semiquantitative fluorescent analysis of 39 different EV surface markers. Figure 3a shows the most relevant markers expressed. EVs within the amniotic fluid of both normal and preeclamptic pregnancies highly expressed tetraspanins, as confirmed with the methods shown above. In addition, the typical amniotic fluid marker, CD24 (Keller et al., 2007) and CD133, known to be highly expressed by urine EVs (Dimuccio et al., 2014) were present at a high level in both EV types, indicating the presence of EVs derived from foetal urine. Expression of the embryonic stem and epithelial cell marker CD326 was detected in both samples. Interestingly, a significantly higher expression of the progenitor/mesenchymal marker SSEA‐4 and CD105 was detected in PE‐EVs (Figure 3a). No expression of leukocyte (CD3, CD4, CD8, CD45, CD56, CD19), endothelial (CD31) or platelet (CD42a and CD49e) markers was observed (data not shown).

ExoView analysis was subsequently used to assess at single‐EV level the expression of NP‐EV and PE‐EV surface markers of interest. After EV affinity binding to tetraspanins, CD105, Tie‐2, CD117 (c‐kit) and HLA‐G expression were evaluated on captured EVs (Figure 3c). The analysis confirmed the presence of HLA‐G, as shown by super‐resolution microscopy, and revealed the presence of the angiogenic marker Tie‐2 and of the stem cell marker CD117 on both NP‐ and PE‐EVs. In addition, the analysis confirmed the increased expression of CD105 in PE‐EVs with respect to NP‐EVs (Figure 3c). By simultaneously detecting HLA‐G along with the other makers, we were able to assess their co‐expression. The analysis showed that the HLA‐G expressing EVs displayed similar CD105 and c‐kit markers levels, suggesting that the increased CD105 expressing EVs present in PE were not of placental origin (Figure 3d). At variance, Tie‐2 levels were significantly lower in placental EVs of PE pregnancies. No differences in tetraspanin levels, used as control, were observed (Supplementary Figure 1B). The increased CD105 expression in PE‐EVs with respect to NP‐EVs was also confirmed by Western Blot analysis (Supplementary Figure 2).

**FIGURE 4:**
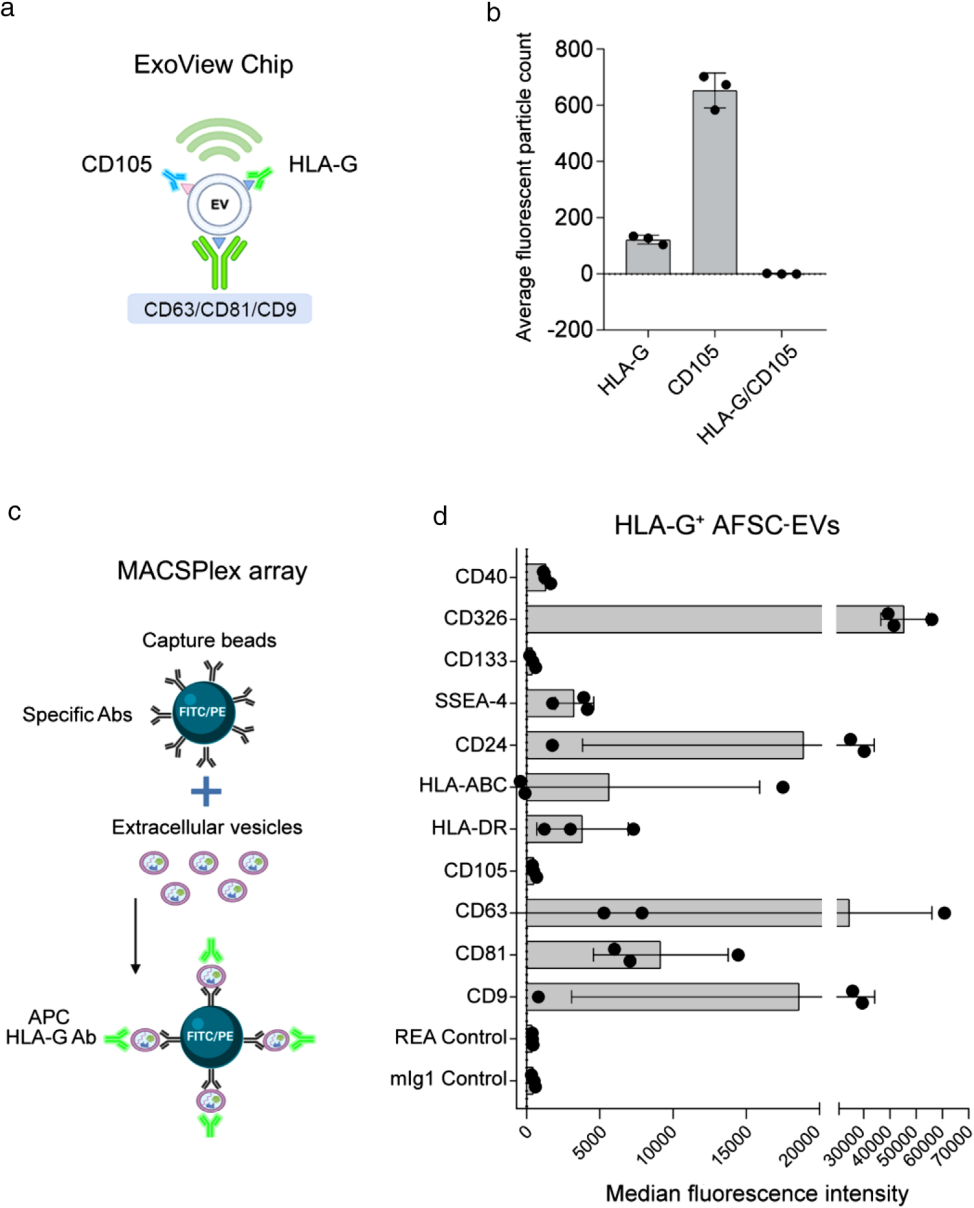
Characterization of HLA‐G‐expressing EVs from term AFSCs. (a) Diagram explaining the experimental method in relation to the graph in panel b. (b) Fluorescent particle count of AFSC‐EVs captured on tetraspanin‐coated chip analysed by ExoView, showing expression of HLA‐G^+^ and CD105^+^ EVs, but lack of co‐expression of the markers. (c) Graphical representation of MASCPlex analyses to characterize HLA‐G^+^ AFSC‐EVs, using Ab‐coated fluorescent beads and APC‐labelled anti‐HLA‐G Ab. (d) The graph shows the median fluorescence intensity of surface markers co‐expressed on HLA‐G^+^ AFSC‐EVs (*n* = 3). AFSC‐EVs (5.8 × 10^8^) were gated for the HLA‐G positivity and analysed for the expression of a panel of markers. No expression of CD105 was detected. Illustrations in panels A and C were created with https://biorender.com

Finally, we tested the characteristics of EVs derived from cultured term AFSCs, as a possible source of amniotic fluid‐EVs (Figure 4). Detection of AFSC‐derived EVs captured on tetraspanin coated chips revealed the presence of distinct subpopulations of HLA‐G and CD105 expressing EVs, being CD105^+^ EVs the larger fraction. No co‐expression of the two markers was observed (Figure 4a and b). Analysis of the HLA‐G^+^ AFSC‐EV fraction by MACSPlex analysis confirmed the lack of CD105 expression (Figure 4c and d). These data suggest that the CD105^+^ EV fraction observed in PE‐EVs could possibly derive from cells of foetal or foetal membrane origin, including AFSCs.

### 3.3 Functional characterization of NP‐EVs and PE‐EVs on angiogenesis

**FIGURE 5:**
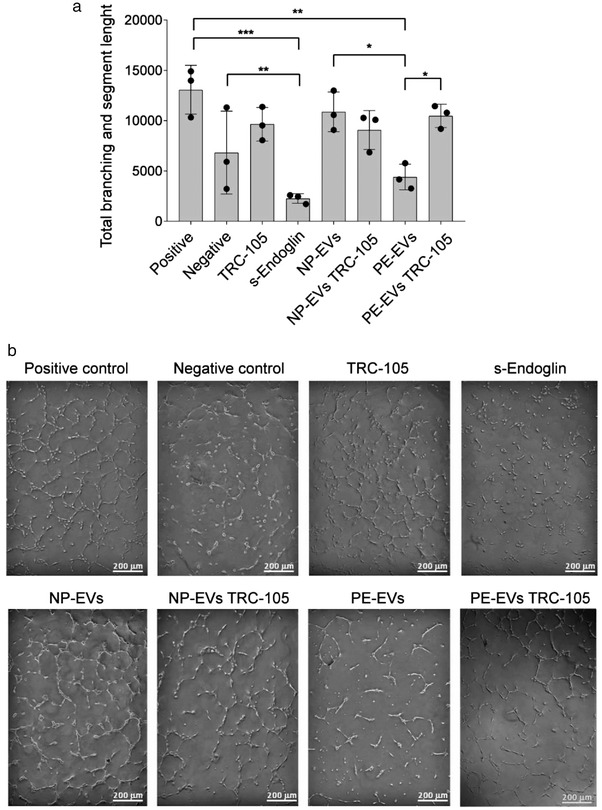
Effect of NP‐EVs and PE‐EVs on tube formation and role of CD105. (a) Quantification of tube lengths of HUVEC treated with NP‐EVs, PE‐EVs with or without anti‐CD105 Ab (TRC‐105) (8 μg/ml). A concentration of 1000 EVs/cell was used. Positive control: complete EBM medium; negative control: EBM medium without FBS. s‐Endoglin (100 ng/ml) was used as control for angiogenesis inhibition. Statistical analysis was performed using ANOVA with Bonferroni's post‐hoc test. * = *P* < 0.05, ** = *P* < 0.01, *** = *P* < 0.001. (b) Representative images of the tube formation assay

Soluble endoglin is a differentially spliced form of endoglin and is a relevant antiangiogenic factor in PE, acting as a decoy receptor for transforming growth factor beta (TGF‐β) (Valluru et al., 2011). To test the angiogenic properties of NP‐EVs, PE‐EVs, and the possible role of CD105 increased expression on the EV surface, a tube formation assay was performed. Amniotic fluid‐derived EVs both from NP and PE, showed a significant reduction in the organization of the HUVEC into capillary‐like structures on Matrigel, with respect to the positive control, with the PE‐EVs being significantly more effective than NP‐EVs in reducing tube formation. To test the relative role of the increased expression level of CD105 on the surface of PE‐EVs, TRC‐105, an anti‐CD105 monoclonal Ab used as an antiangiogenic drug (Brossa et al., 2018), was applied. We found that the addition of TRC‐105 to PE‐EVs, but not to NP‐EVs, prior to HUVEC stimulation significantly abolished their inhibitory effect (Figure 5), indicating the role of surface CD105 in angiogenesis reduction.

## 4 DISCUSSION

**FIGURE 6:**
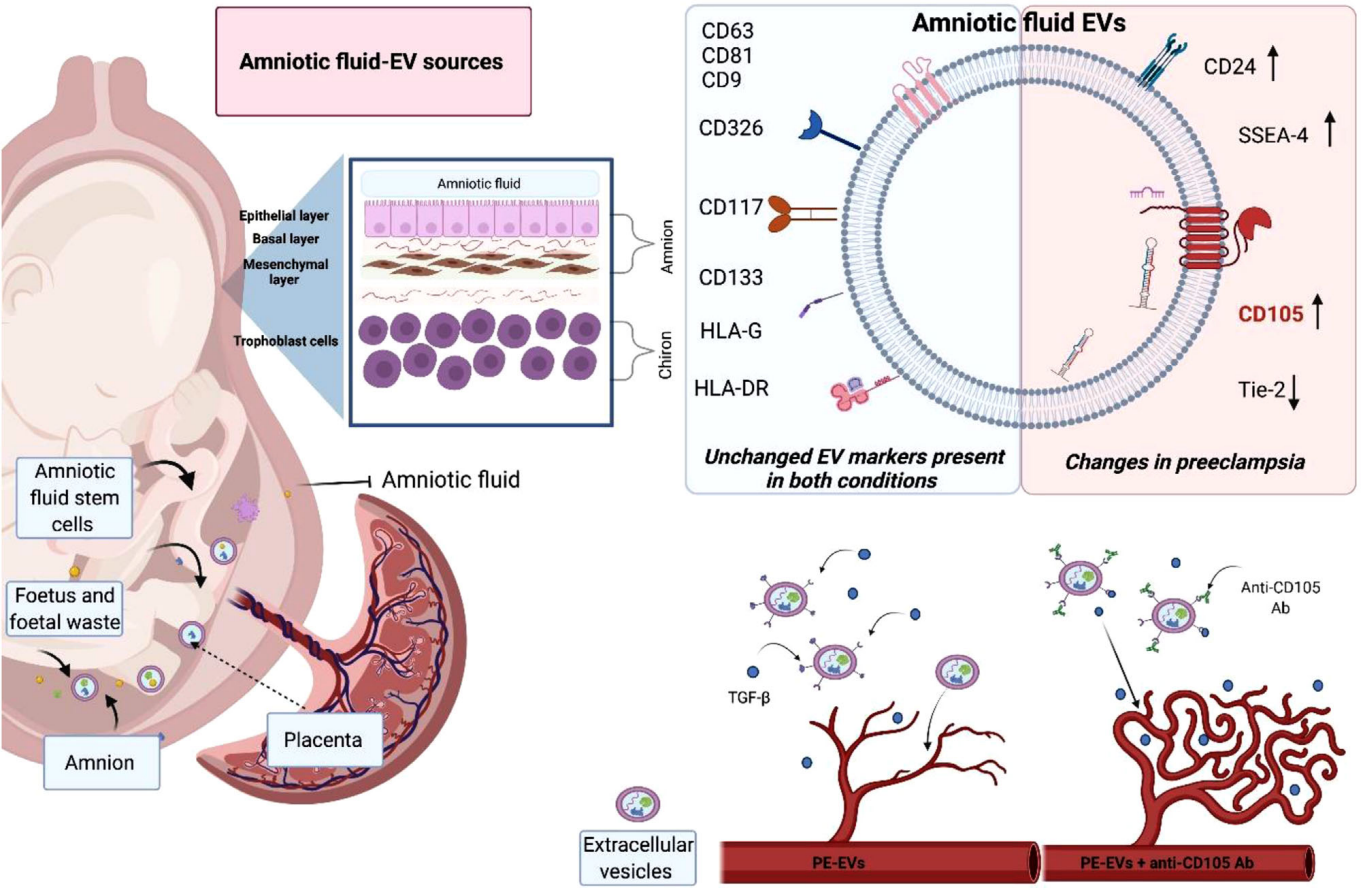
Graphical illustration summarizing the main findings. The figure shows the multiple possible sources of term amniotic fluid‐derived EVs and the main changes in PE‐EVs. Their antiangiogenic properties are supported by the specific upregulation of CD105 surface. The image was created with https://biorender.com

As reproductive EV research advances, investigation of placenta, surrounding gestational tissues, and amniotic fluid is of profound importance, in order to understand the factors involved in foetal development and maternal health. This paper provides for the first time a deep characterization of EVs present in term amniotic fluid in normal and preeclamptic pregnancies. Using several orthogonal techniques (chip‐based platform, cytofluorimetric bead‐based multiplex assay and super‐resolution microscopy analysis), we show that EVs of term amniotic fluid represent a heterogeneous population of EVs of multiple origins, including placental tissues, foetal urine, and stem cells. Moreover, we identified potential antiangiogenic properties of amniotic fluid‐EVs from preeclamptic pregnancies, supported by the specific upregulation of the CD105 surface marker (Figure 6).

We here characterized EVs isolated from term amniotic fluid using sub‐sequential centrifugation, and we further purified them for the identification of specific surface receptors and miRNA cargo using size‐exclusion chromatography to remove contaminating factors possibly present in the amniotic fluid (Monguió‐Tortajada et al., 2019; Théry et al., 2018). The characterization of NP‐EVs at a single‐EV level revealed a highly heterogeneous population of EVs with variable tetraspanins expression. These results are in agreement with those reported by Han and colleagues on colocalization analysis of tetraspanins in EVs derived from different cell lines, showing distinct fractions of single, double or triple co‐expressing EVs (Han et al., 2021).

Moreover, based on surface marker expression, we found that EVs within the term amniotic fluid may originate from various sources. Indeed, amniotic fluid‐EVs expressing CD24 were mainly derived from foetal urine (Keller et al., 2007). In this study, we confirmed the possible cellular origin of the EVs attributed to foetal urine by expression not only of CD24 but also of CD133, which is also considered to be highly present in urinary EVs (Dimuccio et al., 2014). Additionally, amniotic fluid‐EVs expressed mesenchymal and stem markers, possibly suggesting the origin from CD117^+^ amniotic fluid stem cells (Cananzi et al., 2009), amniotic CD105^+^ mesenchymal stem cells (Baghaei et al., 2017), and SSEA‐4^+^ expressing embryonic stem cells (Noisa et al., 2012). Interestingly, we also showed that a large fraction of amniotic NP‐EVs expressed HLA‐G, as detected by ExoView and super‐resolution microscopy. HLA‐G, the distinct HLA isoform involved in maternal tolerance of the fetus, was previously reported to characterize EVs from the placenta in vitro or in vivo in the maternal circulation (Eunsung Mouradian, 2008; Orozco et al., 2009). It is also conceivable that the reported presence of HLA‐G within the amniotic fluid could be related to its expression on the EV membrane (McMaster et al., 1998). The origin of the HLA‐G expressing EVs in the amniotic fluid is unclear. They could possibly directly originate from the amniotic membrane surrounding the fetus and containing the amniotic fluid. Another possible explanation is the passage of trophoblast‐derived EVs through the chorion and amnion to reach the amniotic fluid (McMaster et al., 1998). The placental origin of amniotic fluid‐EVs is also suggested by the analyses of the deregulated miRNA target genes in preeclampsia, showing the most represented cell targets origin from the myoblast, the omentum and the placenta. The placental and stem cell origin of NP‐EVs supports their use in regenerative medicine, as recently proposed (Mitrani et al., 2021).

Proteomic and miRNA profile analysis of amniotic fluid‐EVs has been gaining an increasing interest in identifying disease‐associated biomarkers and mechanisms (Dixon et al., 2018). In this study, we identified the specific increase in the levels of surface‐associated CD105 in PE‐EVs, whereas other surface markers showed similar expression levels. Endoglin (CD105) is a co‐receptor of TGF‐β1 and TGF‐β3, highly expressed on the surface of syncytiotrophoblasts and endothelial cells (Valluru et al., 2011). An increase in soluble endoglin is a hallmark of preeclampsia (Mutter & Karumanchi, 2008; Schuster et al., 2020) and is responsible for endothelial damage by trapping soluble TGF‐β molecules (Valluru et al., 2011). We here confirmed the increased CD105 levels through different orthogonal techniques. The use of a CD105‐based capture chip allowed us to demonstrate that the increase in CD105 expression was not due to HLA‐G expressing placental EVs. The origin of CD105 expressing EVs could be possibly attributed to cells of foetal or foetal membrane origin in the fluid, including AFSCs. Indeed, the analysis of cultured amniotic fluid mesenchymal stromal cells showed that the released EVs were largely CD105^+^, whereas the HLA‐G^+^ EVs, representing a minor fraction, did not co‐express CD105. It would be of interest in future studies to evaluate the CD105/HLA‐G co‐expression by cells isolated from preeclamptic amniotic fluid.

The increased CD105 level in PE‐EVs is in agreement with previous data, showing higher levels of endoglin on EVs produced by placental explants treated with preeclamptic sera (Tannetta et al., 2013) and support the concept that surface receptors expressed by EVs may act as a decoy for soluble factors (Sedrakyan et al., 2017; Tannetta et al., 2013). In analogy, we found that PE‐EVs affected angiogenesis in vitro and that this effect was abolished when CD105 was blocked by a specific antibody. A slight antiangiogenic effect was also observed for NP‐EVs, in parallel with data showing that AFSC‐EVs do not promote angiogenesis despite their regenerative effect (Takov et al., 2020). However, this was not prevented by the anti‐CD105 antibody, suggesting the involvement of other factors. Previous data showed that administration of human EVs derived from preeclamptic placentas in mice resulted in damage to the vasculature, poor foetal nutrition and promoted endothelial permeability and glomeruli damage (Chang et al., 2018). In particular, the study by Chang et al. provided an additional mechanism for the antiangiogenic effect of EVs, related to the presence of soluble endoglin transfer from the EV cargo to endothelial cells (Chang et al., 2018). Our results, showing the antiangiogenic effect of amniotic preeclamptic EVs, highlight a new role for EVs present in the amniotic fluid, possibly acting on both foetal and maternal compartments. Indeed, many reports described feto‐placental endothelial dysfunction in preeclampsia, as a detriment to both the mother and the fetus (Escudero et al., 2016). It would be of interest to investigate the possible effect of PE‐EVs on foetal development.

In conclusion, our study characterizes at single‐EV level, EVs present in the amniotic fluid, an interesting biofluid with a possible relevance for therapeutic application. Amniotic fluid‐derived EVs showed a heterogeneous origin, expressing markers of foetal and placental cells. We highlight the differential expression of antiangiogenic factors in normal and preeclamptic amniotic fluid‐derived EVs, both at surface and cargo level. These characteristics may reflect the hypoxic and antiangiogenic general status of preeclampsia and could possibly play a role by affecting the developing fetus or the surrounding foetal membranes. Finally, EVs within the amniotic fluid could potentially be used as a diagnostic test if investigated at earlier stages of pregnancy.

## CONFLICT OF INTEREST

The authors declare no conflict of interest.

## Supporting information

Supporting Information
