# Supplementary material for: Single extracellular vesicle analysis in human amniotic fluid shows evidence of phenotype alterations in preeclampsia
Source: J Extracell Vesicles. 2022 May 18;11(5):e12217. doi: 10.1002/jev2.12217 (PMC9115584; doi:10.1002/jev2.12217)
Supplement: Supplementary file 1 — Supporting Information [file JEV2-11-e12217-s001.docx]

Single extracellular vesicle analysis in human amniotic fluid shows evidence of phenotype alterations in preeclampsia

Natalia Gebara^1^, Julia Scheel^2^, Renata Skovronova^1^, Cristina Grange^3^, Luca Marozio^4^, Shailendra Gupta^2^, Veronica Giorgione^5^, Federico Caicci^6^, Chiara Benedetto^4^, Asma Khalil^5,7^, Benedetta Bussolati^1*^

1. Department of Molecular Biotechnology and Health Sciences, University of Turin, Italy.
2. Department of Systems Biology and Bioinformatics, University of Rostock, Germany.
3. Department of Medical Sciences, University of Turin, Turin, Italy.
4. Department of Surgical Sciences, Obstetrics and Gynecology, University of Turin, Italy.
5. Vascular Biology Research Centre, Molecular and Clinical Sciences Research Institute, St George's University of London, UK
6. DiBio Imaging facility, University of Padua, Italy.
7. Fetal Medicine Unit, St George's University Hospitals NHS Foundation Trust, St George's University of London, UK.

^*^**Corresponding Author:** Benedetta Bussolati, Molecular Biotechnology Centre, University of Turin, via Nizza 52, 10126 Turin, Italy. Fax 011-6331184, Tel. 011-6706453, e-mail: [benedetta.bussolati@unito.it](about:blank)

***Supplementary Table 1.*** *Volume of amniotic fluid and the corresponding concentration of EVs as particles/ml of initial volume, based on nanoparticle tracking analysis.*

|  | Normal pregnancies | | Preeclampsia | |
| --- | --- | --- | --- | --- |
| Sample N. | Sample volume (ml) | Concentration (particles/ml) | Sample volume (ml) | Concentration  (particles/ml) |
| 1 | 20 | - | 12 | 2.80E+09 |
| 2 | 10 | 4.5 E+09 | 46 | 3.48E+09 |
| 3 | 6 | 2.58E+09 | 11 | 1.13E+09 |
| 4 | 12 | 2.81E+09 | 15 | 2.21E+09 |
| 5 | 4 | 3.8E+09 | 15 | 1.03E+09 |
| 6 | 4 | 3.6E+09 | 15 | 5.02E+09 |
| 7 | 10 | 2.53E+09 | 15 | 3.30E+09 |
| 8 | 4 | 6.59E+08 |  |  |
| 9 | 19.5 | 2.75E+09 |  |  |
| 10 | 4.5 | 2.18E+09 |  |  |
| 11 | 22 | 3.18E+11 |  |  |
| 12 | 15 | - |  |  |
| 13 | 22 | 3.08E+09 |  |  |
| 14 | 21 | 2.61E+09 |  |  |
| 15 | 20 | 3.08E+09 |  |  |
| 16 | 10 | 4.5E+09 |  |  |
| 17 | 6 | 2.58E+09 |  |  |
| 18 | 12 | 2.81E+09 |  |  |
| 19 | 4 | 3.8E+09 |  |  |
| 20 | 4 | 3.6E+09 |  |  |
| 21 | 19 | 2.79E+09 |  |  |
| 22 | 15 | - |  |  |
| 23 | 19 | 3.65E+09 |  |  |
| 24 | 22 | 2.27E+09 |  |  |
| 25 | 10 | 4.5E+09 |  |  |
| 26 | 6 | - |  |  |
| 27 | 30 | - |  |  |
| 28 | 15 | 2.27E+09 |  |  |
| 29 | 19 | 4.9E+08 |  |  |
| 30 | 19 | 2.98E+09 |  |  |
| 31 | 15 | 2.27E+09 |  |  |
| 32 | 19 | 2.81E+09 |  |  |
| 33 | 22 | - |  |  |
| 34 | 10 | - |  |  |
| 35 | 25 | 6.776E+10 |  |  |
| 36 | 18 | 5.481E+10 |  |  |
| 37 | 22.5 | - |  |  |
| 38 | 41 | 3.914E+11 |  |  |
| 39 | 27.5 | - |  |  |
| 40 | 15 | - |  |  |

*
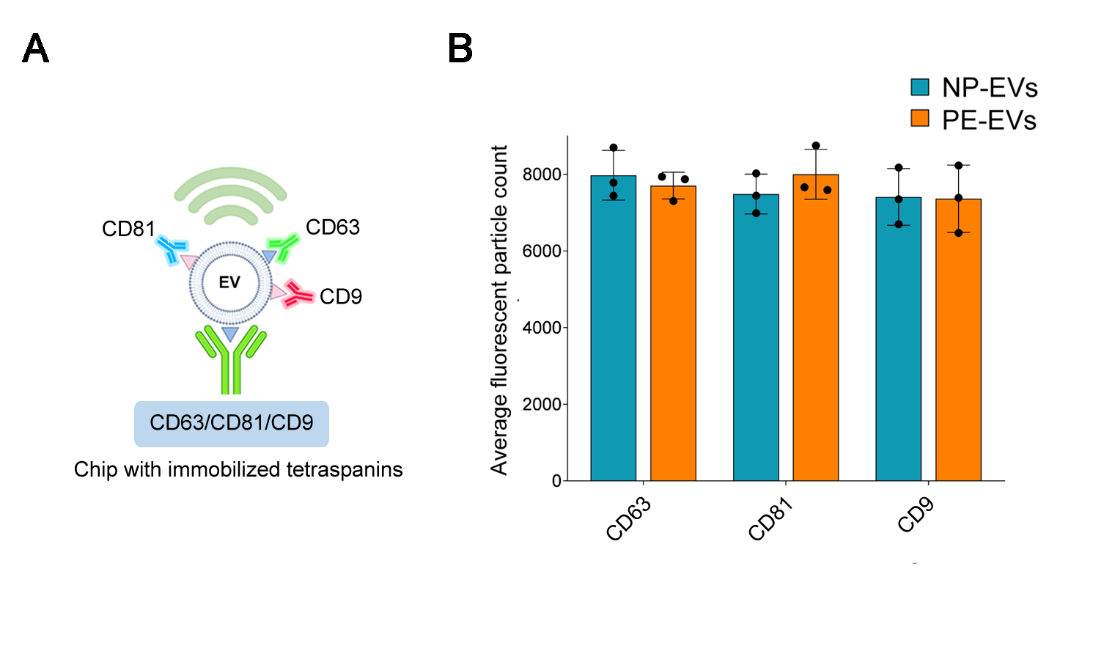
*

***Supplementary Figure 1****.* ***ExoView analysis of amniotic fluid-derived NP-EVs (n=3) and PE-EVs (n=3).*** *5.8x10^8^ EVs in final volume of 35 µl of buffer were used for all samples.* ***A)*** *Image in panel B was created with* [*https://biorender.com*](https://biorender.com)*.* ***B)*** *ExoView chips spotted with single tetraspanins and detected with anti- CD63, CD81, CD9 fluorescent Abs confirmed a similar tetraspanins expression level between NP and PE-EVs. Three different samples were analysed for each experimental condition. No statistical differences were observed.*


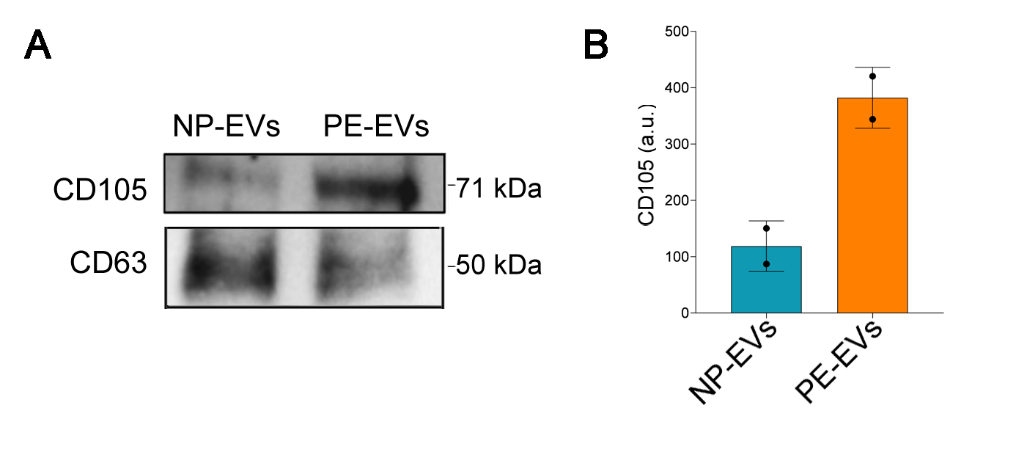


***Supplementary Figure 2****. Western Blot image and band intensity quantification showing CD105 expression differences between NP-EVs and PE-EVs.* ***A****) Representative image of the Western Blot showing the presence of CD105 and CD63 in NP- and PE-EVs.* ***B)*** *Quantification of Western Blot. CD63 was used as a loading control and the total lane intensity of CD105 was normalized to CD63 and used for the protein analysis. Results of two different analyses, each using pools of three different NP-EV or PE-EV samples, are shown.*
